# Blindness in a Sri Lankan woman with bilateral breast lumps: a case report

**DOI:** 10.1186/s13256-015-0792-4

**Published:** 2015-12-29

**Authors:** Vipula R. Bataduwaarachchi, Rukshani Galappaththi, Nirmali Tissera

**Affiliations:** 1 Department of Pharmacology and Pharmacy, Faculty of Medicine University of Colombo, PO Box 271, Kynsey Road, Colombo 08, Sri Lanka; 2 Department of Medicine, National Hospital, Ward Place, Colombo 07, Sri Lanka

**Keywords:** Granulomatous breast lump, Granulomatosis with polyangiitis, Hemorrhagic retinal angiopathy, Rituximab

## Abstract

**Background:**

Granulomatosis with polyangiitis is a rare multisystemic autoimmune disorder predominantly affecting the upper and lower respiratory tracts and the kidneys, and rarely affecting other organ systems. Tuberculosis can mimic the presentation of granulomatosis with polyangiitis, and both can occur simultaneously in the same patient. Here we report what we believe to be the first case of concurrent granulomatous breast lesions and hemorrhagic retinal angiopathy in a Sri Lankan woman with refractory granulomatosis with polyangiitis complicated by probable tuberculosis.

**Case presentation:**

A 48-year-old Sri Lankan Moorish woman presented with a 6-month history of ulcerating bilateral breast lumps, a 3-month history of non-healing painful ulcers on the palate, and sudden bilateral painless loss of vision. Retinoscopy confirmed left-sided retinal hemorrhages and bilateral panuveitis. An examination of her respiratory system showed bilateral coarse crepitations. Histologic examination of the palatal and breast lesions showed chronic granulomatous inflammation. Her levels of immune markers were elevated but her renal function was normal. Chest radiography showed bilateral mid-zone and lower-zone infiltrates with cavitation and small pleural effusions. Her serum proteinase 3 anti-neutrophil cytoplasmic antibody titer and the level of adenosine deaminase in her pleural fluid were significantly elevated. She was diagnosed with generalized granulomatosis with polyangiitis complicated with probable pulmonary tuberculosis, and was started on methylprednisolone and cyclophosphamide pulse therapy with anti-tuberculous treatment. She later developed cerebral vasculitis, indicating refractory disease, and was treated with second-line rituximab with excellent response.

**Conclusion:**

Proteinase 3 anti-neutrophil cytoplasmic antibody may be a valuable diagnostic marker in patients with atypical symptoms of granulomatosis with polyangiitis or in the presence of probable tuberculosis. Retinal vascular angiopathy needs to be diagnosed and treated early to prevent the development of complete blindness. Concomitant cytotoxic and anti-tuberculous treatments may be safe and effective in patients with simultaneous refractory disease with probable tuberculosis.

## Background

Granulomatosis with polyangiitis (GPA), also known as Wegener’s granulomatosis, is a rare multisystem autoimmune disorder predominantly affecting the upper and lower respiratory tracts and the kidneys [1]. It has a spectrum of clinical presentations, and new manifestations may appear during the course of the disease. Necrotizing granulomatous inflammation and vasculitis of small and medium blood vessels are characteristics of this disorder. Proteinase 3 anti-neutrophil cytoplasmic antibody (PR3-ANCA) is strongly associated with GPA, and over 90 % of patients have been reported to demonstrate ANCA positivity during active disease [2]. About 30 cases of breast granulomatosis have been reported in association with GPA to date, but a concurrent association with hemorrhagic retinal angiopathy has not been reported [3–6]. GPA can affect any part of the eye, and one previous study described the case of a patient presenting with hemorrhagic retinal angiopathy as the first clinical sign [7]. Tuberculosis (TB) can mimic the pulmonary symptoms of GPA, and their simultaneous occurrence can thus lead to diagnostic confusion and consequent management challenges.

Here, we report what we believe to be the first case of concurrent granulomatous breast lesions and hemorrhagic retinal angiopathy, which occurred in a Sri Lankan woman with refractory GPA complicated with probable pulmonary TB.

## Case presentation

**Fig. 1:**
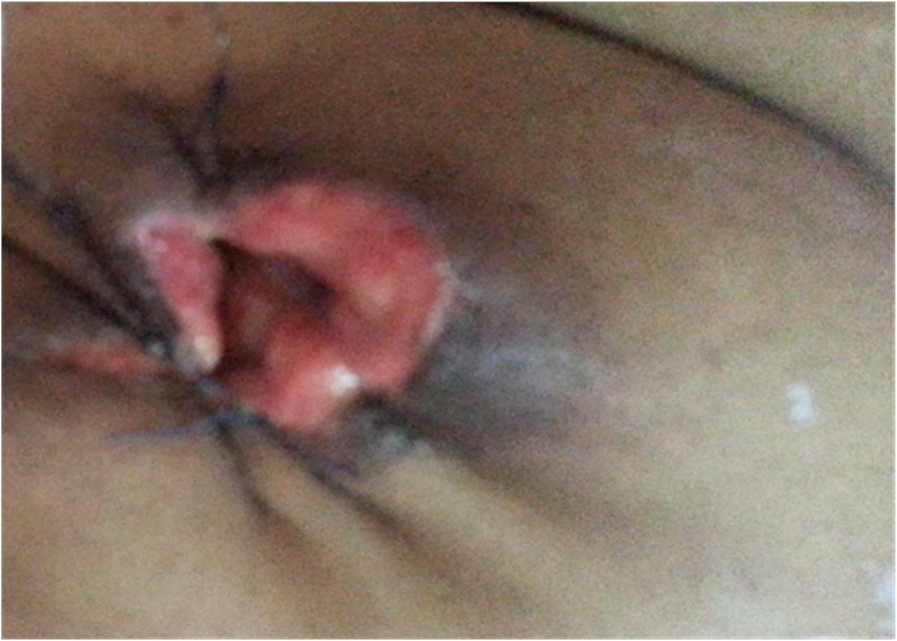
Appearance of the right breast after wound cleaning. A large ulcer is visible destroying the nipple and areola. Sutures were placed to oppose the gaping edges of the wound. A few granulomatous whitish papules are visible projecting out from the subcutaneous tissues

**Fig. 2:**
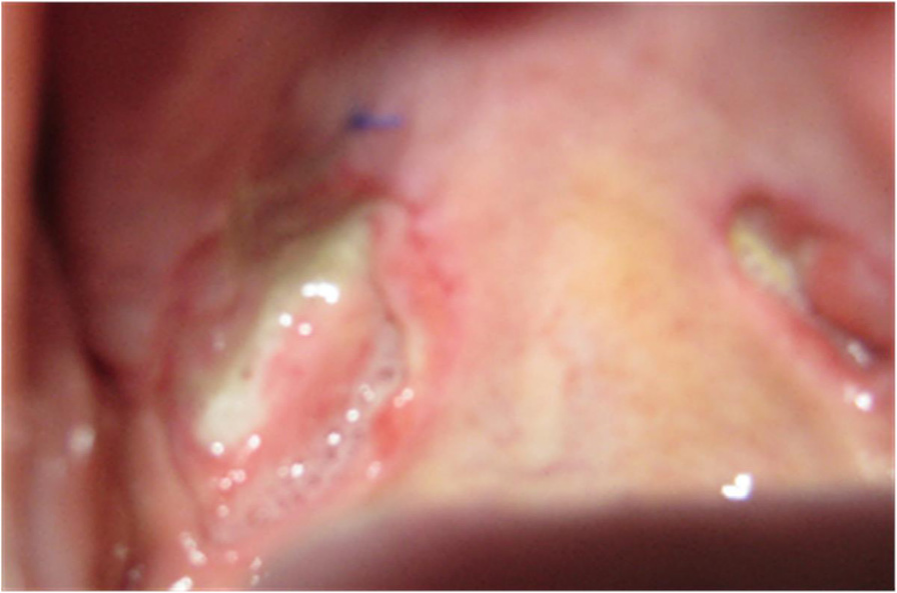
Appearance of the palate after wound cleaning and biopsy. A large ulcer (1.5 × 1.3 cm) with a sharp edge and unhealthy base covered with slough is seen at the margin between the soft and hard palates on the right side. A smaller similar ulcer is present on the left side. A suture was placed at the biopsy site

A 48-year-old Sri Lankan Moorish woman from Colombo presented to our emergency treatment unit with bilateral sudden-onset painless loss of vision. There was no associated tearing, irritation, or red eyes. Six months previously she had noted bilateral, slowly growing breast lumps for which she has not taken medical advice, on cultural grounds. The lumps subsequently became ulcerated, with intense pain and discomfort (Fig. 1). She also complained of painful non-healing ulcers in her palate over the previous 3 months, with no associated anogenital ulceration (Fig. 2). Background constitutional symptoms had been present for 1 year, but the results of the rest of her systemic review were normal. After admission, she developed a dry cough and moderate hemoptysis without fever. She had no family or contact history of TB, and no family history of malignancies or autoimmune disorders.

A general examination revealed that our patient was of average build with no lymphadenopathy. She did not consent to a genital examination. An examination of her respiratory system showed bilateral diffuse coarse crepitations. There was no dullness over her lung fields and no bronchial breathing was present. Her skin and nails appeared normal with no stigmata of chronic disease. Results from cardiovascular, rheumatological, and neurological examinations were normal.

### Visual assessment

**Fig. 3:**
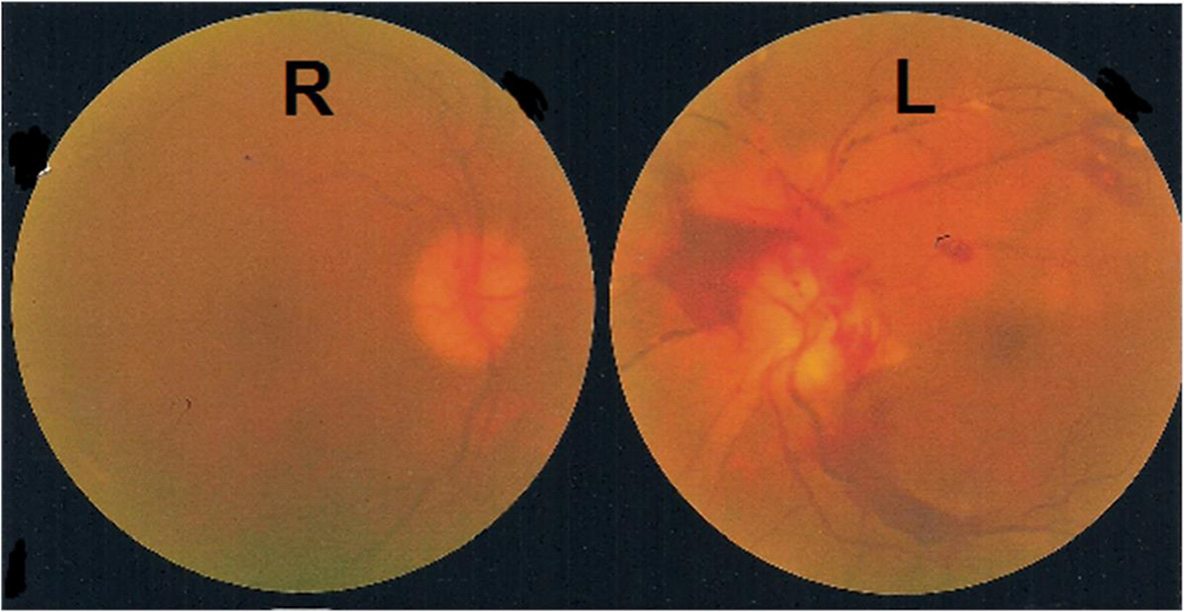
Indirect retinoscopy image at presentation. A large retinal hemorrhage is present around the left optic disc. Pre-retinal retrohyaloidal hemorrhages and segmental perivascular changes are also visible

Our patient’s light perception was absent bilaterally and she had no direct or indirect light reflexes, suggesting a bilateral visual pathway lesion. Results from magnetic resonance imaging of her brain and orbits were normal, excluding any gross abnormality of the posterior visual pathway. Indirect retinoscopy showed retinal hemorrhage due to retinal vascular angiopathy in her left retina with bilateral panuveitis (Fig. 3). Visual evoked potentials were unrecordable on both sides, further supporting this diagnosis.

### Laboratory assessment

**Fig. 4:**
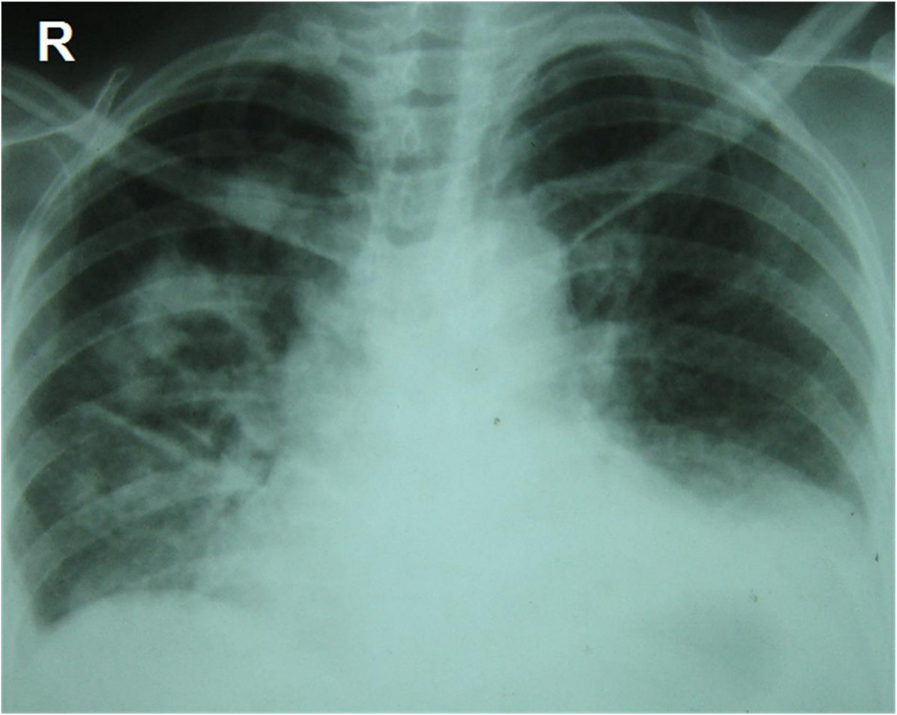
Chest radiography at presentation. Multiple nodular and cavitating lesions are visible predominately on the right side with multiple patchy infiltrates typical of granulomatosis with polyangiitis. Costophrenic angles are obliterated owing to small bi-basal pleural effusions

**Fig. 5:**
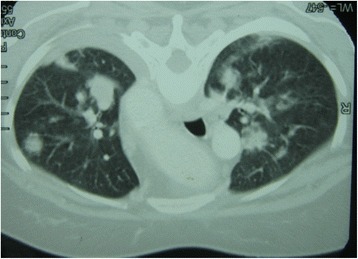
Contrast-enhanced thoracic computed tomography at presentation. Multifocal alveolar infiltrates are visible

Our patient’s immune markers were elevated, with an erythrocyte sedimentation rate of 135 mm/h and C-reactive protein level of 96 mg/dL. Chest radiography showed bilateral mid-zone and lower-zone infiltrates with cavitation and small pleural effusions (Fig. 4). Contrast-enhanced thoracic computed tomography confirmed the presence of multifocal alveolar infiltrates, cavitations, and effusions (Fig. 5). A Mantoux test was not performed because she had undergone one previously, though the results were not available. Although a γ-interferon-release assay would have been useful, we did not have the facilities to perform this investigation. A sputum acid-fast bacilli (AFB) test was negative in three consecutive samples. The difference between her serum and pleural fluid protein levels was 15 g/L and the differential lymphocyte count was 70 %. Her lactate dehydrogenase level was 600 U/L in her pleural fluid. Results from cytology, Gram staining, AFB, and TB polymerase chain reaction and cultures of the pleural fluid were negative.

An ultrasound scan of her breast showed multiple hypoechoic ill-defined areas suggestive of abscesses. A biopsy of the abscess wall showed well-formed granulomas with collections of histiocytes and multinucleated giant cells. There was no caseation to indicate TB or cytological abnormalities suggestive of malignancy. Histologic examination of the palatal ulcers indicated a similar pattern of granulomatous inflammation with some necrotic areas. Screening for human immunodeficiency virus was negative and her normal levels of angiotensin-converting enzyme excluded sarcoidosis. Her renal function and results of a full urine report were normal. However, her serum PR3-ANCA titer was significantly elevated at 190.74 IU/mL (normal <20 IU/L) although her myeloperoxidase-neutrophil cytoplasmic antibody, anti-nuclear antibody, and rheumatoid factor titers were all normal. Her pleural fluid adenosine deaminase (ADA) level was significantly elevated at 101 IU/L (normal <2 IU/mL).

### Diagnosis

The pulmonary and oral manifestations together with strong positivity for PR3-ANCA confirmed the diagnosis of generalized GPA in our patient [8]. This was associated with rare manifestations of breast granulomatosis and hemorrhagic retinal angiopathy. However, renal involvement, which indicates a poor prognosis, was absent [9]. Significantly elevated pleural fluid ADA together with cavitating lung lesions suggested probable concurrent TB. TB commonly causes upper lobe lung cavitation, compared with mid-zone and lower-zone cavitations that are more common in GPA. Our patient refused to have a pleural/lung biopsy, which would have provided histological and/or microbiological confirmation of TB. According to the consultant chest physician’s opinion, we considered probable coexisting TB based on her markedly high pleural fluid ADA levels. Initiation of cytotoxic treatment presented a management dilemma in our patient because of the presence of organ-threatening GPA.

### Therapy and follow-up

Our patient was started on intravenous methylprednisolone (1,000 mg/day for 3 days) and intravenous cyclophosphamide at a dose of 15 mg/kg every 2 weeks for the first three pulses, followed by infusions every 3 weeks for the next 3–6 pulses, as per European League Against Rheumatism recommendations [10]. She was also started on full anti-tuberculous therapy (ATT) with rifampicin 5 mg/kg, isoniazid 10 mg/kg, pyrazinamide 30 mg/kg, and ethambutol 15 mg/kg with pyridoxine, as per World Health Organization guidelines. Her pulmonary, breast, and oral lesions slowly improved, but her vision did not recover. Her liver and renal functions were closely monitored. After completing 2 months of treatment she presented with sudden-onset generalized tonic clonic convulsions. Brain magnetic resonance imaging indicated multiple infarctions, suggesting possible cerebral vasculitis, though results from a magnetic resonance arteriogram and magnetic resonance venogram were normal. Tests for anti-phospholipid antibodies were negative, excluding the possibility of coexisting anti-phospholipid syndrome. Refractory disease was considered and she was started on second-line rituximab 375 mg/m^2^ by intravenous infusion once weekly for 4 weeks, with intravenous methylprednisolone 1,000 mg/day for 3 days followed by prednisone 1 mg/kg per day [11, 12]. Aspirin 75 mg was started daily. Complete clinical remission was achieved, though her vision did not recover, probably because of permanent damage to the retina. She continued with ATT for 6 months on the basis of probable TB, with resolution of the pulmonary lesions. Our patient was referred to a rehabilitation program to address her blindness.

## Discussion

We believe this is the first reported case of concurrent granulomatous breast lesions and vasculitis-like hemorrhagic retinal angiopathy in a patient with GPA. The presence of ADA-positive cavitating lung lesions suggested probable TB, even though this was not confirmed by microbiological tests. Given that the radiological pattern of the pulmonary lesions was indicative of GPA, in the absence of a tissue diagnosis our patient was considered to have coexisting pulmonary TB on the basis of pleural fluid ADA. The prevalence of TB in urban Sri Lanka (13.9 %) is higher than in rural areas (2.2 %) [13], possibly associated with poor housing conditions, especially in some areas in Colombo. It was therefore reasonable to consider the probability of TB in this patient. Even though TB can present with chronic oral ulceration and breast granulomatosis, histologic examination of the lesions from our patient showed well-formed granulomas with multinucleate giant cells and histiocytes, in contrast to the caseation seen in TB.

The presence of blindness and organ-threatening GPA created a management challenge in terms of the timing of cytotoxic treatment in our patient. We opted to treat both conditions simultaneously, with close monitoring of organ function. The appearance of a new symptom complex of multiple infarctions on magnetic resonance imaging raised the possibility of treatment for refractory disease. Our patient showed an excellent clinical response to second-line rituximab in the absence of renal involvement. The recent RITUXVAS trial, which was conducted among patients with newly diagnosed GPA or microscopic polyangiitis with renal involvement, found no difference between the rituximab and control groups, while B-cell return was associated with relapse [14].

Although TB can be associated with elevated serum PR3-ANCA levels, the histology in the current case was more indicative of GPA than TB [15]. The interpretation of elevated ADA levels in the absence of a tissue diagnosis is controversial, and ATT with immunosuppression is justifiable. In our case, it resulted in a marked clinical and radiological response [16]. Previous results regarding concomitant ATT and cytotoxic treatment in similar presentations further support this treatment strategy in our patient [17, 18]. The prognosis for patients treated with rituximab for rheumatoid arthritis in the presence of TB has been shown to be excellent, though the current case is the first to report the use of rituximab with appropriate ATT cover in a patient with both TB and refractory GPA [19]. Further studies are needed to determine the long-term outcomes and optimal treatment regimens to be used under similar circumstances.

## Conclusions

Although GPA affecting the breast is rare, it should nonetheless be considered in the evaluation of any breast mass. Retinal vascular angiopathy needs to be diagnosed and treated early to prevent the development of complete blindness. A full systemic evaluation is thus essential in patients diagnosed with GPA. Sarcoidosis, immune deficiency, and disseminated malignancy should be excluded in a patient presenting with breast lumps; pulmonary and ocular lesions, the nature of the lesions, histological results, and biomarkers are important for making a differential diagnosis in such cases. PR3-ANCA is a valuable diagnostic marker in patients with atypical symptoms of GPA or concurrent similar illness such as TB. Simultaneous cytotoxic treatment, including rituximab, and ATT can be safe and effective in patients with refractory GPA and probable TB.

## Consent

Written informed consent was obtained from the patient for publication of this case report and any accompanying images. A copy of the written consent is available for review by the Editor-in-Chief of this journal.

## Abbreviations

ADA: Adenosine deaminase
AFB: Acid fast bacilli
ATT: anti-tuberculosis treatment
GPA: granulomatosis with polyangiitis
PR3-ANCA: proteinase 3 anti-neutrophil cytoplasmic antibody
TB: tuberculosis
